# Toward a multidisciplinary approach to the study of tick-borne diseases

**DOI:** 10.3389/fcimb.2014.00118

**Published:** 2014-09-30

**Authors:** Agustín Estrada-Peña, José de la Fuente García

**Affiliations:** ^1^Animal Health (Parasitology), University of ZaragozaZaragoza, Spain; ^2^Biology and Health, IRECCiudad Real, Spain; ^3^Department of Animal Pathology, University of OklahomaStillwater, OK, USA

**Keywords:** ticks, tick-transmitted pathogens, review literature as topic, ecology, epidemiology

“The biology and ecology of ticks shape the potential for the transmission of zoonotic pathogens” is a collection of research and review articles related to the study of tick-borne diseases, with a focus on the methodology to explore the basic relationships between the tick-transmitted pathogens and the environment. It is well-known that a multidisciplinary point of view is necessary in order to develop a global vision of this growing problem. Therefore, in order to promote this holistic approach to the knowledge of tick-borne diseases, this research topic, which will be assembled as an e-book, contains collaborations of entomologists, epidemiologists, virologists, parasitologists, bacteriologists, zoologists, molecular biologists, and veterinarians. As stated in the title, the book addresses some of the factors that are behind the emergence and/or reemergence of tick-borne diseases. Contributions include, among others, interesting reviews on the immune system of ticks, the dissemination of pathogens by bird-carried ticks, an assessment of the main methodological gaps in the research of tick ecology and the epidemiology of tick-transmitted pathogens, the control of tick-borne pathogens by anti-tick vaccines, or reviews about prominent tick-transmitted diseases.

It is now well-known that several climatic, environmental, and sociodemographic changes that have occurred over the past years are behind the resurgence of some tick transmitted pathogens worldwide. Other than the impact on the animal production systems and the derived problems in the husbandry, there are serious concerns about how tick-transmitted pathogens would affect human and animal health. Global change, defined as the impact of human activity on the fundamental mechanisms of biosphere functioning, includes not only climate change, but also habitat transformation, water cycle modification, biodiversity loss, and synanthropic incursion of alien species into new territories (Gortázar et al., 2014). All these factors may affect the ecological relationships of ticks with their hosts and the microorganism community and pathogens they carry (Estrada-Peña et al., 2014). We thus opted to present here compilations and reviews regarding some of the most important tick-transmitted pathogens, together with some facts regarding tick as a system such as tick stress response that have been overlooked in other reviews.

As editors of “The biology and ecology of ticks shape the potential for the transmission of zoonotic pathogens,” we would like to acknowledge all coauthors for their valuable and interesting contributions and we wish the readers of this e-book a productive and enjoyable reading of some of the most innovative work related to tick-borne diseases.

## Conflict of interest statement

The authors declare that the research was conducted in the absence of any commercial or financial relationships that could be construed as a potential conflict of interest.
